# Advanced aggressive clinical features may be associated with immune dysfunction in patients with HIV-positive Hodgkin lymphoma in the cART era: a multicenter study from China

**DOI:** 10.3389/fimmu.2025.1628909

**Published:** 2025-09-03

**Authors:** Xiping Liang, Chaoyu Wang, Yifeng Tang, Xiaoqing Xie, Yan Wu, Haiyan Min, Wei Zhang, Wenwen Zhou, Vishnu Prasad Adhikari, Xiaomei Zhang, Yao Liu

**Affiliations:** ^1^ Chongqing University Cancer Hospital, Chongqing Key Laboratory of Translational Research for Cancer Metastasis and Individualized Treatment, Chongqing, China; ^2^ Henan Infectious Disease Hospital, The Sixth People’s Hospital of Zhengzhou, Zhengzhou, Henan, China; ^3^ Yunnan Provincial Hospital of Infectious Diseases, Kunming, Yunnan, China; ^4^ Peking Union Medical College Hospital, Chinese Academy of Medical Sciences (CAMS) & Peking Union Medical College, Beijing, China; ^5^ Department of Hepatobiliary Surgery & Pancreatic Surgery, The First Affiliated Hospital, Zhejiang University, Hangzhou, China

**Keywords:** HIV, HL, CD4, immune function, survival

## Abstract

**Introduction:**

Combination antiretroviral therapy (cART)-mediated immune reconstitution can establish a tumor-permissive microenvironment. In addition, compromised immune surveillance may contribute to more aggressive disease phenotypes in HIV patients; however, clinical evidence remains limited.

**Methods:**

We conducted a retrospective analysis of clinical data of newly diagnosed Hodgkin lymphoma (HL) patients from 2014 to 2024 treated at four medical centers in China. The authors conducted clinical and immune function analysis of HIV-positive HL patients with special emphasis on prognosis and immune factors.

**Results:**

In total, 19 patients were diagnosed as HIV positive. HIV-positive HL patients (HIV-HL) had more advanced stage disease, ECOG-PS, bulky disease, and B symptoms compared to HL patients without HIV (n=130). HIV-positive HL patients had decreased CD4 cell count, CD4/CD8, and GZMB. Lower CD4 count was associated with more bulky disease and B symptoms and higher IL-2R and IL-6 levels in HIV-HL patients. And HIV-HL patients with bulky disease had less GZMB compared to non-bulky disease patients. The enrichment impact of gene alterations on bulky disease demonstrated that PI3K/AKT, thyroid hormone signaling, NF−kappa B signaling pathway, and EBV infection were involved. Immune dysfunction (CD4, CD8, and CD4/CD8), on the other hand, showed no association with survival in both HIV-positive and negative HL patients. There were similar outcomes in patients with and without HIV treated by ABVD chemotherapy.

**Conclusion:**

HIV-associated Hodgkin lymphoma (HIV-HL) often presents with more aggressive clinical features, although outcomes are similar to those observed in HIV-negative HL patients. Impaired immune function may contribute to an increased tumor burden through multiple mechanisms. However, it was not associated with outcomes. HL treatment approaches might not necessarily require adjustment solely due to HIV status, but additional clinical evidence is needed to support this assertion.

## Introduction

Since the introduction of combined antiretroviral therapy (cART), the incidence of opportunistic infections and AIDS-defining malignancies, such as Kaposi sarcoma (KS) and invasive cervical cancer, has declined among people living with HIV (PLWH). In contrast, the incidence of Hodgkin lymphoma (HL)—a non-AIDS-defining cancer—has remained stable or even increased in this population in recent decades (1, 2). Despite advances in HIV management, PLWH continue to face a higher overall cancer risk compared to the HIV-negative population (3). Specifically, PLWH have a 5- to 26-fold increased risk of developing HL, with those receiving cART experiencing an even greater risk (20- to 30-fold higher) (4–6).

The pathogenesis of this elevated risk may be attributed to cART-mediated immune reconstitution establishing a tumor-permissive microenvironment. Specifically, the expansion of CD4+ T-cell populations following cART initiation may paradoxically provide trophic support for Reed-Sternberg cells—the pathognomonic malignant cells of HL—while simultaneously compromising immune surveillance through multiple mechanisms (7). Molecular profiling reveals that HIV-associated HL demonstrates distinct oncogenic signatures, including enhanced EBV-driven oncoprotein expression and aberrant NF-κB pathway activation, correlating with more aggressive disease phenotypes compared to HIV-negative HL cases (8, 9).

Current clinical evidence reveals a therapeutic paradox in PLWH: although modern HL treatment protocols have achieved comparable complete response (CR) rates in HIV-positive and HIV-negative patients, significant disparities in progression-free survival (PFS) and overall survival (OS) outcomes persist across different cohorts. While some studies report a 1.5-2-fold increase in treatment failure rates among PLWH, other well-controlled investigations, including the Botswana trial, have demonstrated equivalent 5-year survival outcomes (10, 11). To elucidate these clinical discrepancies and better characterize the biological and prognostic determinants of HIV-associated HL within global epidemiological contexts, we conducted a multidimensional analysis of Chinese PLWH, incorporating clinical, molecular, and therapeutic outcome parameters.

## Methods

### Patients and clinical data analysis

We performed a multicenter retrospective cohort analysis of newly diagnosed HL patients who received care at four tertiary medical centers in China between December 2014 and December 2024. Participating institutions were The Sixth People’s Hospital of Zhengzhou, Peking Union Medical College Hospital, Yunnan Provincial Hospital of Infectious Diseases, and Chongqing University Cancer Hospital. All cases underwent centralized pathological review by at least two board-certified hematopathologists to confirm the diagnosis.

### Data collection and ethical considerations

We systematically extracted demographic characteristics, clinical manifestations, laboratory parameters, and treatment details from electronic medical records. The study protocol adhered to the ethical principles of the revised Helsinki Declaration (2014) and received approval from all participating institutions’ ethics committees. Written informed consent was obtained from all enrolled patients.

### Diagnostic and staging procedures

Diagnostic confirmation followed the 2016 WHO classification criteria for hematolymphoid neoplasms, requiring both characteristic morphological features and definitive immunohistochemical profiles. Clinical staging was performed according to the 2014 Lugano staging criteria, based on the extent of disease involvement and the presence of B symptoms. B symptoms included 1) unexplained fever and a temperature >38 °C for more than 3 consecutive days, excluding infection; 2) night sweats (soaking clothes); and 3) weight loss >10% of body weight within the six months prior to diagnosis. Patients were divided into groups with or without B symptoms based on their clinical presentation.

### Treatment and response assessment

All patients received the ABVD regimen (doxorubicin, bleomycin, vinblastine, and dacarbazine): on days 1 and 15, patients received 25 mg/m^2^ doxorubicin, 10 mg/m^2^ bleomycin, 3 mg/m^2^ vinblastine, and 375 mg/m^2^ dacarbazine. The median number of chemotherapy cycles was six (ranging from two to 20). HIV-infected patients were administered cART. This included two nucleoside reverse transcriptase inhibitors and one nonnucleoside reverse transcriptase inhibitor. ^18^F-fluorodexyglucose positron emission tomography/computed tomography (PET/CT) was performed for radiological evaluation. Treatment response was primarily assessed according to the 2014 Lugano criteria, classified as CR, partial response (PR), stable disease (SD), or disease progression (PD).

### Immunostaining and GZMB test

The tissues were sectioned at 4μm and processed for immunostaining according to the manufacturer’s instruction of the Absin five-color multiple fluorescent immunohistochemical staining kit (Absin, abs50013-20T). The following primary antibodies were used: Anti-Granzyme B antibody (Abcam, ab255598), Anti-CD4 antibody (A, ab288724), Rabbit anti-CD8 Monoclonal Antibody (Absin, abs171445), CD19 Polyclonal antibody (Proteintech, 27949-1-AP), and CD68 Monoclonal antibody (Proteintech, 66231-2-Ig). All the immunostained sections were imaged by the Leica confocal microscope.

### Enrichment analysis

We implemented pathway enrichment analysis with the Metascape website. Gene Ontology (GO) as well as the Kyoto Encyclopedia of Genes and Genomes (KEGG) were used as references, and functional enrichment analysis of recurrently altered genes was conducted using clusterProfiler (v4.0) in R. Significantly altered genes (mutated in ≥15% samples, q<0.05) were tested against the KEGG 2021 database with Fisher’s exact test. Results were FDR-corrected, and terms with FDR<0.05 were visualized as enrichment maps.

### Follow-up

Follow-up was conducted by reviewing inpatient medical records and telephone interviews, with a follow-up cutoff date of December 31, 2024, and a median follow-up duration of 83 months (ranging from 4 to 257 months). PFS was defined as the time interval from HL diagnosis to disease progression, death, or the last follow-up. OS was defined as the time interval from HL diagnosis to patient death or the last follow-up.

### Statistical analysis

Statistical analysis was performed using SPSS 22.0 software, and graphs were generated using Graph Pad Prism Version 9.0.0. Continuous data were presented as median (range), and categorical data were presented as frequencies (%). Chi-square and fisher’s exact test were used appropriately according to sample sizes. Interquartile range (IQR) was used for the test of inflammatory cytokines. Survival analysis was conducted using the Kaplan-Meier method.

## Results

### Patient characteristics

Among the 19 HIV-positive HL patients, 17 were male, with a median age of 43 years (range: 22–74 years). Of the cases, 17 were of the mixed cellularity subtype and two were of the lymphocyte-predominant subtype. In seven cases, HIV infection was discovered at the time of lymphoma diagnosis and standard antiretroviral therapy was commenced immediately upon HIV diagnosis. Among the remaining 12 cases, the median time from HIV diagnosis to lymphoma development was 22.2 months (range, 4.3-56.4 months). Patients diagnosed before 2020 received a combination of two nucleoside reverse transcriptase inhibitors and one non-nucleoside reverse transcriptase inhibitor for antiretroviral therapy, while those diagnosed after 2020 were treated with bictegravir/emtricitabine/tenofovir alafenamide (Biktarvy). In terms of disease staging, four cases were in stage III and 15 cases were in stage IV. Five patients had an Eastern Cooperative Oncology Group performance status (ECOG PS) score >1, and eight had elevated lactate dehydrogenase (LDH) levels. One patient had central nervous system involvement, five had bone marrow involvement, six had large masses (with the longest tumor diameter >7.5 cm), and 16 patients presented with B symptoms. The median CD4 T cell count (Normal rang 550-1440×10^6/L) at lymphoma diagnosis was 110 × 10^6/L (range, 24-434 × 10^6/L), with five cases having CD4 T cell counts <50 × 10^6/L. The median HIV viral load was 50 copies/mL (range: 0-87202×10^6/L), with five cases having viral loads below measurable limits. Among the patients, 12 had concomitant Epstein-Barr virus (EBV) infection and one had hepatitis B virus (HBV) infection. In terms of safety, the combination of cART and ABVD chemotherapy did not exacerbate adverse reactions. The patient characteristics are summarized in **Table 1**.

### Comparison between HIV-positive and negative HL patients

HIV-HL patients are more likely to present with mixed cellularity type (89.5% VS 43.1%, p=0.004) and be male (89.5% VS 68.5%, P=0.046) compared to HL without HIV patients. Compared to non-HIV-infected HL patients, HIV-HL patients exhibited a higher prevalence of adverse prognostic factors, including more advanced stage (100% VS 52.3%, p<0.001), worse ECOG-PS (26.3% VS 2.3%, p=0.001), bulky disease (26.3% VS 3.8%, p=0.003), B symptoms (84.2% VS 33.8%, p<0.001), and bone marrow involvement (26.3% VS 5.4%, p=0.009). There were no significant different characteristics in age, Epstein-Barr virus, CNS involvement, or chemotherapy regimen (P>0.05) (shown in **Table 1**).

In order to know the immune function, a comparison was made between HIV-positive and negative patients. Comparative analysis of immune parameters revealed that HIV-HL patients had significantly reduced median values in multiple hematologic and immunologic markers, including CD4+ cells (110 cells/μL vs. 483 cells/μL; p<0.001) and lower CD4/CD8 ratio (31.6% vs. 16.2%, p<0.001). HIV-positive HL patients were also more likely to have cytopenia, including WBC decrease (42.1% VS 16.2%, p=0.008), lymphopenia (79% VS 35.4%, p<0.001), neutropenia (31.6% VS 6.2%, p p<0.001), and thrombocytopenia (31.6% VS 11.5%, p=0.019))than the HIV-negative controls (shown in **Table 1**).

**Table 1:** Baseline clinical characteristic of HL patients.

Characteristic	Total (N=149) (%)	HIV positive (N=19)/(%)	HIV negative (N=130)/(%)	p-Value
Demographics				0.046
Male	106(71.1)	17(89.5)	89(68.5)	
Female	43(28.9)	2(10.5)	41(31.5)	
Age, years, mean (range)	43(8-84)	43(22-74)	43(8-84)	0.199
≤60	122(81.9)	18(94.7)	104(80)	
>60	27(18.1)	1(5.3)	26(20)	
HIV factors				-
HIV-transmission category			-	-
MSM	10/19(52.6)	10(52.6)	–	–
Heterosexuals	9/19(47.4)	9(47.4)	–	–
Concomitant HIV and lymphoma diagnosis	7/19(36.8)	7(36.8)	–	–
HIV viral load, copies/mL, median (range)	50 (0–87202)	50 (0–87202)	–	–
Undetectable patients	5/19(26.3)	5(26.3)	–	–
CD4 count, ×10^6^ /L, median (range)	454(24-1686)	110(24-434)	483(47-1686)	<0.001
<50×10^6^ /L	7(4.7)	5(26.3)	2(1.5)	
(50-200) ×10^6^ /L	19(12.8)	8(42.1)	11(8.5)	
>200×10^6^ /L	123(82.6)	6(31.6)	117(90)	
CD4/CD8, below normal (<0.71)	33(22.1)	12(63.2)	21(16.2)	<0.001
Concurrent EBV	61/138(44.2)	12(63.2)	49/119(41.2)	0.062
ART	19/19(100)	19(100)	–	–
No ART at diagnosis	7/19(36.8)	7(36.8)	–	–
Previous ART resistance	12/19(63.2)	12(63.2)	–	–
Lymphoma factors				
Lymphoma subtype				0.004
Mixed cellularity	73(49.0)	17(89.5)	56(43.1)	
Lymphocyte predominant	27(18.1)	2(10.5)	25(19.2)	
Nodular sclerosis	26(17.4)	0	26(20)	
Lymphocyte depletion	1(0.7)	0	1(0.8)	
Unclassified	22(14.8)	0	22(16.9)	
ECOG PS >1	8(5.4)	5(26.3)	3(2.3)	0.001
Stage 3–4	87(58.4)	19(100)	68(52.3)	<0.001
WBC (Normal: 4-10×10^9/L)				0.008
4-10×10^9/L	120(80.5)	11(57.9)	109(83.8)	
<4×10^9/L	29(19.5)	8(42.1)	21(16.2)	
Lymphocyte				<0.001
Normal (1.1-3.2×10^9/L)	88(59.1)	4(21.0)	84(64.6)	
Decrease	61(40.9)	15(79.0)	46(35.4)	
Neutrophils				<0.001
Normal (1.8-6.3×10^9/L)	135(90.6)	13(68.4)	122(93.8)	
Decreased	14(9.4)	6(31.6)	8(6.2)	
PLT				0.019
Normal (125-350×10^9/L)	128(85.9)	13(68.4)	115(88.5)	
Decreased	21(14.1)	6(31.6)	15(11.5)	
CD4/CD8				0.031
Normal (0.71-2.87)	121(81.2)	12(63.2)	109(83.8)	
Decreased	28(18.8)	7(36.8)	21(16.2)	
Elevated B2-MG (>2.3mg/L)	80(53.7)	18(94.7)	62(47.7)	<0.001
Elevated LDH (>250U/L)	40(26.8)	8(42.1)	32(24.6)	0.095
CNS involvement	1(0.7)	1(5.3)	0	0.128
Bone marrow involvement	12(8.1)	5(26.3)	7(5.4)	0.009
Bulky disease	11(7.4)	6(31.6)	5(3.8)	0.003
B symptoms	60(40.3)	16(84.2)	44(33.8)	<0.001
Treatment factors				
Chemotherapy regimen				
ABVD	149(100)	19(100)	130(100)	0.240
CNS prophylaxis	1(0.7)	1(5.3)	–	0.128
ART use	19/19(100)	19(100)	–	–
Concurrent ART with chemotherapy	19/19(100)	19(100)	–	–

**Figure 1:**
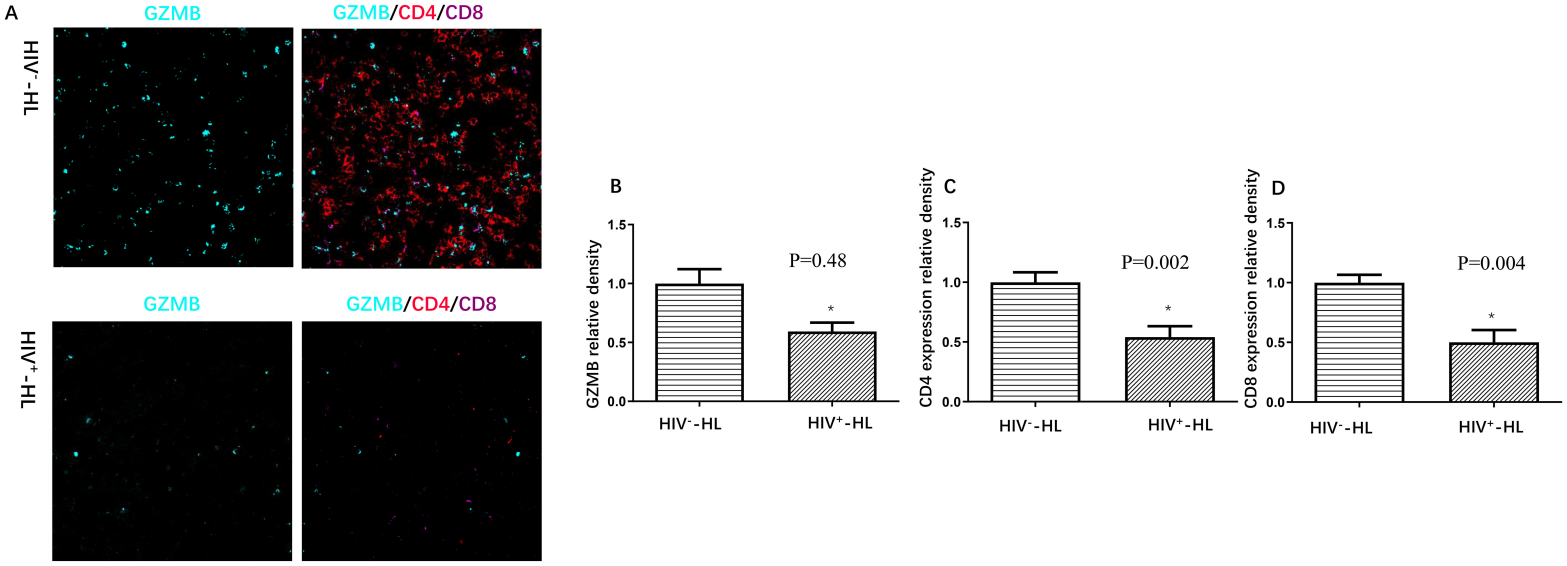
HIV-positive patients (n=6) present worse immune function **(A)**, including less GZMB **(B)**, less CD4 expression **(C)** and less CD8 expression **(D)** when compared to HIV-negative cases (n=6). *P<0.05 VS HIV-negative patients.

In order to know the immune function in HIV-HL patients, we used Granzyme B (GZMB), which plays a pivotal role in immune response, to test the immune cytotoxicity in immune cells. The cytotoxic activity of immune cells was assessed using multicolor immune cell staining. Compared to HIV-negative patients, HIV-positive patients had less GZMB (p=0.48), CD4 (p=0.002) and CD8 (P=0.004) (shown in **Figure 1**). This suggests that HIV-HL patients are more likely to present with advanced clinical symptoms and reduced immune cell counts.

### The clinical features with different CD4 cells in HIV-positive HL patients

As is well known, HIV primarily targets CD4 T cells, resulting in lower CD4 T cell counts compared to the general population. Although cART aids in the recovery of CD4 T cells, they often do not reach normal levels. In the cohort of HIV-HL, a CD4 count below 200 cells/μL was used as the baseline. Among the HIV-positive HL patients, patients were stratified based on the CD4 cell count, where 13/19(68.4) and 6/19(31.6) were in the below 200 and above 200 cohort, respectively (shown in **Table 2**). Patient characteristics were similar between the two cohorts, including gender, age, concurrent Epstein-Barr virus, HIV transmission route, cART therapy, years of HIV infection prior to lymphoma, ECOG-PS, WBC, PLT, CD4/CD8, elevated serum lactate dehydrogenase (LDH), elevated serum β2-microglobulin (β2-MG), Ann Arbor stage, extra-nodal involvement, bone marrow involvement, and chemotherapy regimen (p>0.05). However, the immunocompromised cohort (CD4 ≤ 200 cells/μL) exhibited a distinct clinical profile. Mixed cellularity-HL was the predominant histologic subtype (100% vs 66.7%, p=0.028), accompanied by cytopenia (lymphopenia: 92.3% vs 50%, p=0.035; neutropenia: 46.2% vs 0% p=0.044), advanced tumor burden (bulky lymphadenopathy: 38.5% vs 0%, p=0.032), and systemic symptoms (B symptoms: 100% vs 50%, p=0.005) compared to CD4 > 200 cells/μL patients. The full clinical characteristics are summarized in **Table 2**. The findings suggested that lower CD4 T cell count was more likely to be related to advanced tumor burden.

**Table 2:** Baseline clinical characteristics of HIV positive HL patients.

Characteristics	Total (N=19)(%)	CD4≤200 (N= 13)/(%)	CD4>200 (N = 6)/(%)	p-Value
Demographics				0.310
Male	17(89.5)	11(84.6)	6(100)	
Female	2(10.5)	2(15.4)	0	
Age, years, mean (range)				0.485
≤60	18(94.7)	12(92.3)	6(100)	
>60	1(5.3)	1(7.7)	0	
HIV factors				
HIV-transmission category				0.405
MSM	10(52.6)	6(46.2)	4(66.7)	
Heterosexuals	9(47.4)	7(53.8)	2(33.3)	
Concomitant HIV and lymphoma diagnosis	7(36.8)	3(23.1)	4(66.7)	0.067
Concurrent Epstein-Barr virus	12(63.2)	8(61.5)	4(66.7)	0.829
ART				0.067
No ART at diagnosis	7(36.8)	3(23.1)	4(66.7)	
Previous ART resistance	12(63.2)	10(76.9)	2(33.3)	
Lymphoma factors				
Lymphoma subtype				0.028
Mixed cellularity	17(89.5)	13(100)	4(66.7)	
Lymphocyte predominant	2(10.5)	0	2(33.3)	
ECOG PS >1	5(26.3)	4(30.8)	1(16.7)	0.516
Stage 3–4	19 (100)	13(100)	6(100)	
WBC (Normal: 4-10×10^9/L)				0.127
4-10×10^9/L	11(57.9)	6(46.2)	5(83.3)	
<4×10^9/L	8(42.1)	7(53.8)	1(16.7)	
Lymphocyte				0.035
Normal (1.1-3.2×10^9/L)	4(21.0)	1(7.7)	3(50.0)	
Decreased	15(79.0)	12(92.3)	3(50.0)	
Neutrophils				0.044
Normal (1.8-6.3×10^9/L)	13(68.4)	7(53.8)	6(100)	
Decreased	6(31.6)	6(46.2)	0	
PLT				0.241
Normal (125-350×10^9/L)	13(68.4)	10(76.9)	3(50.0)	
Decreased	6(31.6)	3(23.1)	3(50.0)	
CD4/CD8				0.067
Normal (0.71-2.87)	12(63.2)	10(76.9)	2(33.3)	
Decreased	7(36.8)	3(23.1)	4(66.7)	
Elevated B2-MG (>2.3mg/L)	18(94.7)	13(100)	5(83.3)	0.130
Elevated LDH (>250U/L)	8(42.1)	5(38.5)	3(50.0)	0.723
Bone marrow involvement	5(26.3)	5(38.5)	0	0.636
Bulky disease	6(31.6)	6(46.2)	0	0.032
B symptoms	16(84.2)	13(100)	3(50.0)	0.005
Treatment factors				
Chemotherapy regimen				
ABVD	19(100)	13(100)	6(100)	–
CNS prophylaxis	1(5.3)	0	1(16.7)	0.130
ART use	19(100)	13(100)	6(100)	–
Concurrent ART with chemotherapy	19(100)	13(100)	6(100)	–

**Figure 2:**
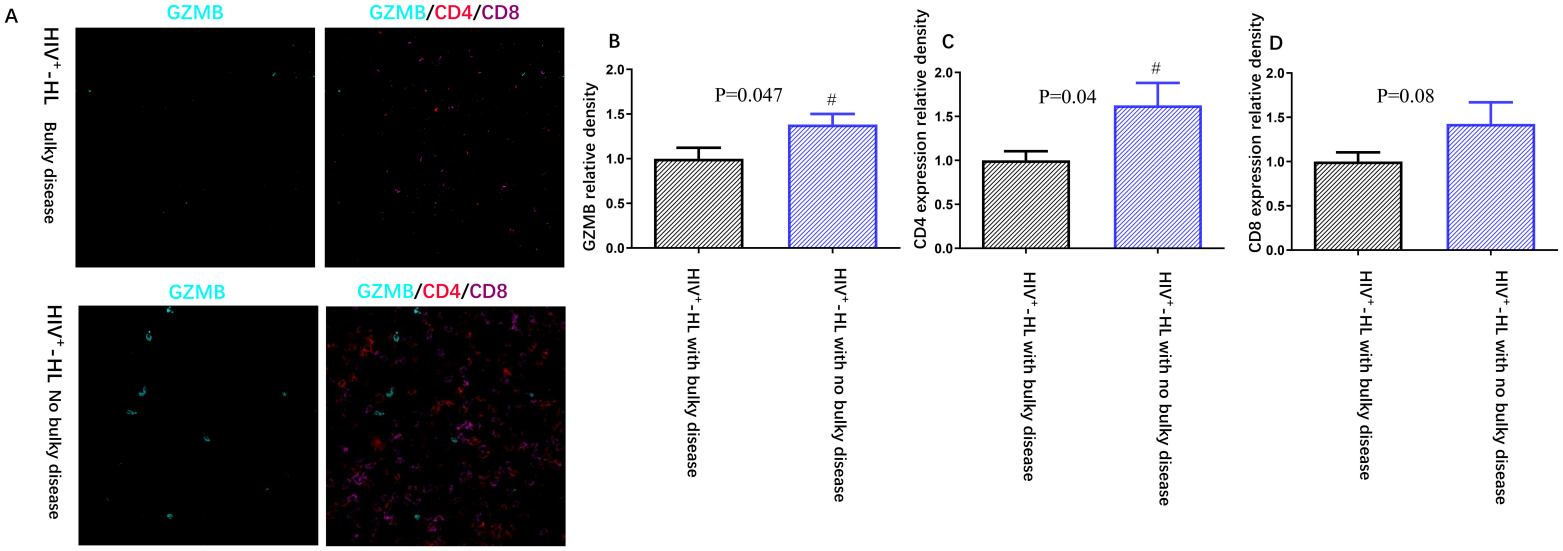
HIV-positive patients (n=6) present worse immune function **(A)**, including less GZMB **(B)**, less CD4 expression **(C)** and less CD8 expression **(D)** when compared to HIV-negative cases (n=6). *P<0.05 VS HIV-negative patients.

In order to know the association between tumor burden and immune function in HIV-HL patients, we tested the expression of GZMB in HIV-HL patients with or without bulky disease. Our result show that the bulky disease cases demonstrated less GZMB (p=0.047) and less CD4 (p=0.04) than HIV-negative without bulky cases (shown in **Figure 2**). However, there was no significate difference in CD8 T cell expression in HIV patients with or without bulky disease. Patients with HIV exhibit elevated levels of interleukin-2 receptor (IL-2R) and interleukin-6 (IL-6) in circulation compared to HIV-negative patients (p=0.02, p=0.04, respectively). This dysregulation is particularly pronounced in HIV-HL patients presenting with bulky disease, who demonstrate significantly higher IL-2R (p=0.04) and IL-6 (p=0.03) compared to HIV-positive patients without bulky tumor burden (**Table 3**).

**Table 3:** The secretion of inflammatory cytokines of HL patients.

Inflammatory factors	HL patients (IQR)			HIV positive (IQR)		
	HIV positive N=12	HIV negative N=24	p	Bulky disease N=6	No Bulky disease N=6	p
IL-1β	6.45 (7.79)	10.45 (18.67)	0.67	6.25 (8.77)	9.78 (15.56)	0.78
IL-2	0.55 (0.78)	0.61 (0.89)	0.56	0.60 (0.59)	0.62 (0.79)	0.80
IL-2R	1.54 (0.51)	1.02 (1.25)	0.02	1.62 (0.56)	0.99 (1.55)	0.04
IL-4	3.23 (2.55)	3.45 (3.27)	0.76	3.34 (3.56)	3.05 (3.12)	0.82
IL-5	1.26 (2.37)	1.18 (3.56)	0.85	1.22 (2.13)	1.01 (2.95)	0.65
IL-6	1.34 (1.47)	0.69 (0.66)	0.04	1.43 (1.24)	0.75 (0.89)	0.03
IL-8	720.5 (1180.3)	756.4 (2100.7)	0.33	690 (1201.5)	745.4 (1990.6)	0.45
IL-10	0.68 (0.58)	0.86 (0.83)	0.51	0.72 (0.62)	0.77 (0.93)	0.56
TNF-α	0.77 (1.59)	1.39 (1.72)	0.58	0.78 (2.16)	1.35 (1.85)	0.64
IFN-γ	12.6 (78.83)	12.34 (25.66)	0.88	11.9 (70.55)	13.55 (19.66)	0.38

**Figure 3:**
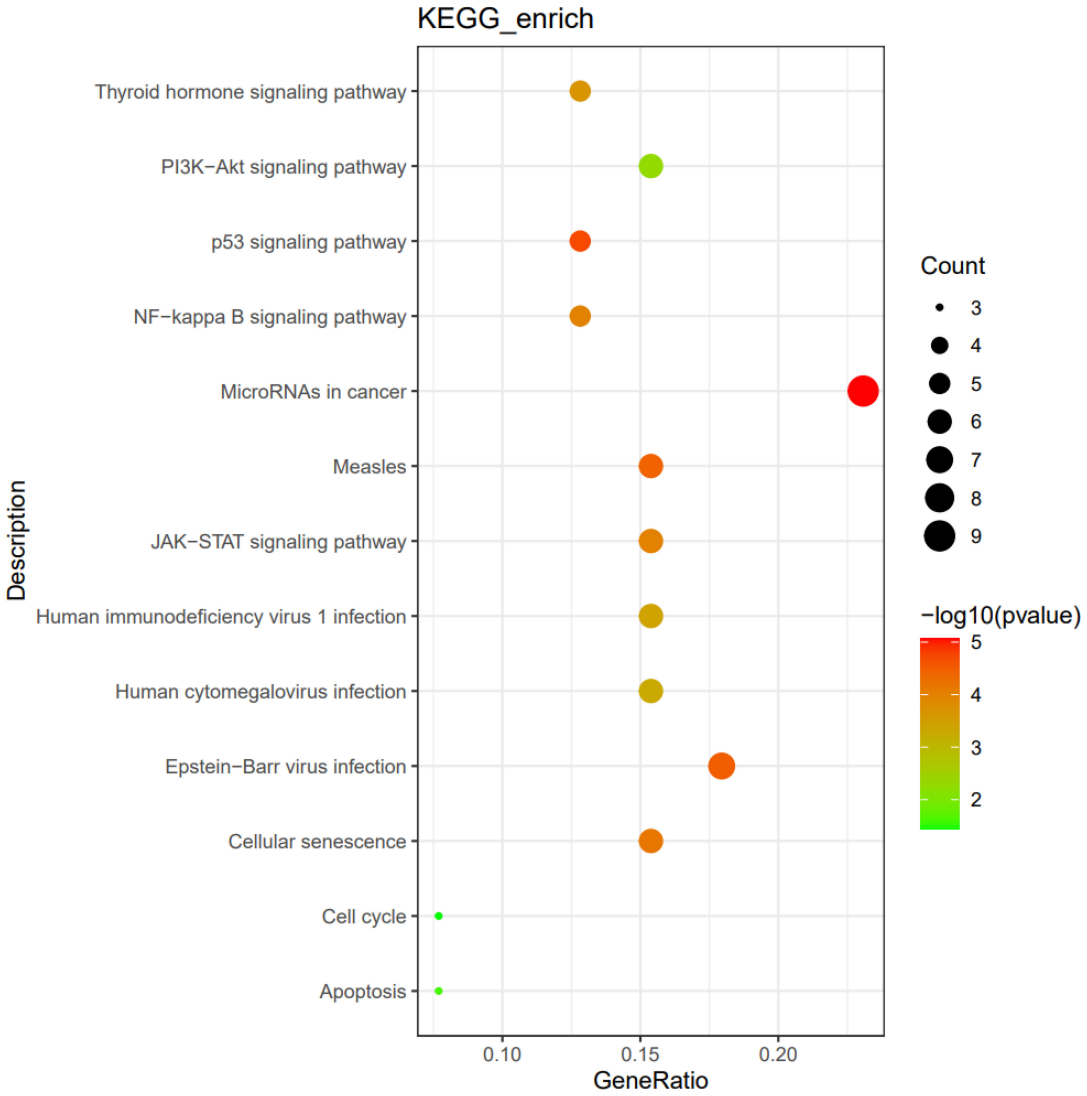
The enrichment impact of gene alterations on pathways in HIV-positive patients with bulky disease (n=6).

We further assessed the possible mechanisms of bulky disease in HIV-positive patients. The enrichment impact of gene alterations on pathways was explored, including the PI3K/AKT pathway, thyroid hormone signaling pathway, NF−kappa B signaling pathway, and EBV infection (shown in **Figure 3**).

### Survival of HL patients

ABVD was the standard chemotherapy protocol for general HL patients. Regardless of HIV infection, all 149 patients received the ABVD regimen. Among these, 77 (51.7%) patients achieved CR and 72 (48.3%) achieved PR, resulting in an overall response rate (ORR) of 100%. Among the HIV-infected HL patients, nine patients (47.4%) achieved CR and 10 (52.6%) achieved PR, resulting in an overall response rate (ORR) of 100%. Two died due to severe infections during treatment, while the remaining 17 HIV patients survived.

**Figure 4:**
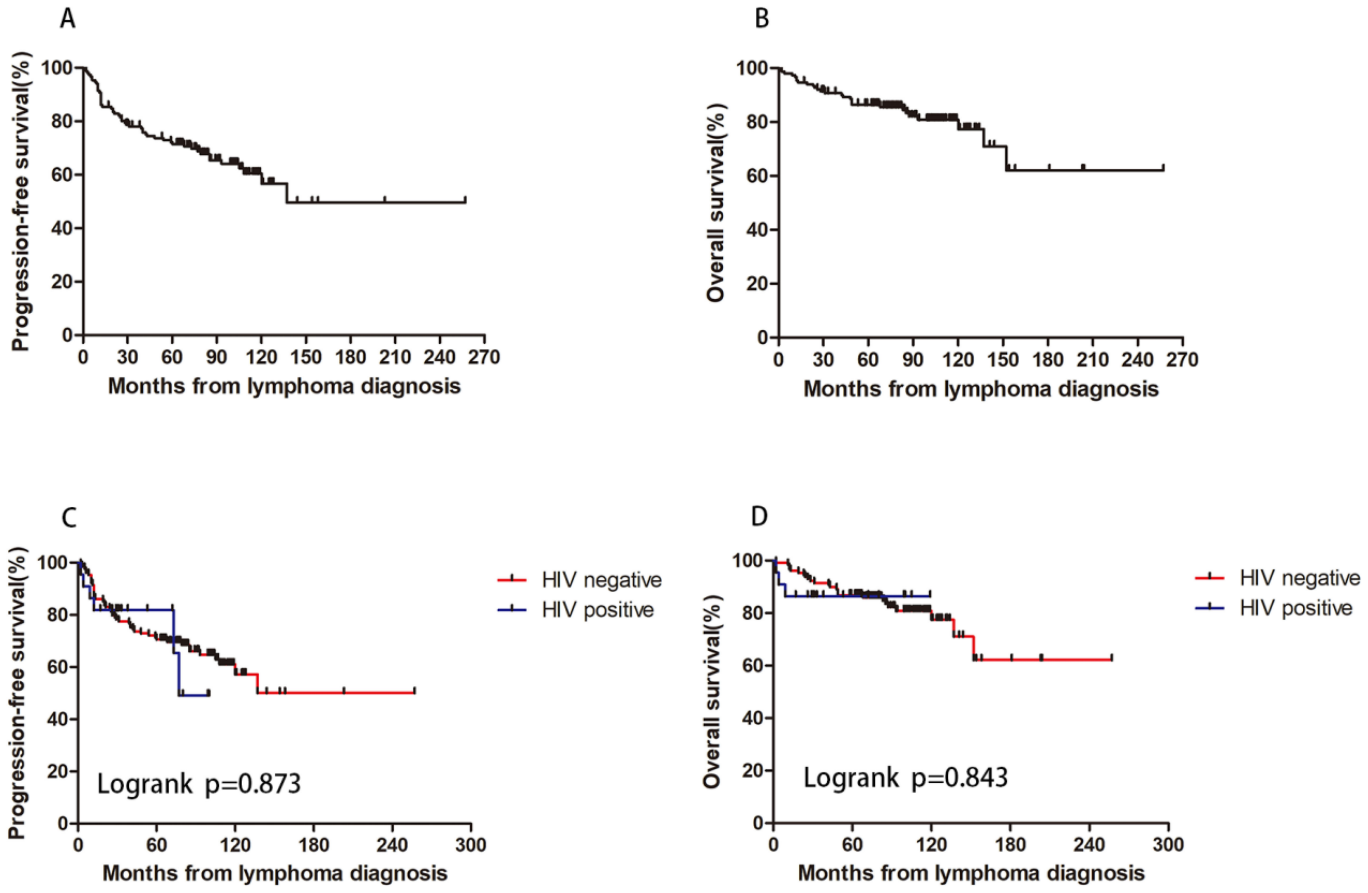
HIV infection and patients’ survival. With a 5-year PFS rate of 71.5% **(A)** and a 5-year OS rate of 86.4% **(B)**. The 5-year PFS rate were 70.5% and 81.8% for HIV negative and positive patient (p=0.873) **(C)**. The 5-year OS rate were 86.8% and 86.4% for HIV negative and positive patient (p=0.843) **(D)**.

The median follow-up duration for the patients was 83 months (range: 4 to 257 months), with a five-year PFS rate of 71.5% and a five-year OS rate of 86.4%. The five-year PFS rates were 70.5% for HIV-negative patients and 81.8% for HIV-positive patients (p=0.873). The five-year OS rates were 86.8% for HIV-negative patients and 86.4% for HIV-positive patients (p=0.843) (**Figure 4**). These results show that, under first-line ABVD therapy and cART for HIV patients, the survival for HL patients was similar regardless of HIV status.

**Figure 5:**
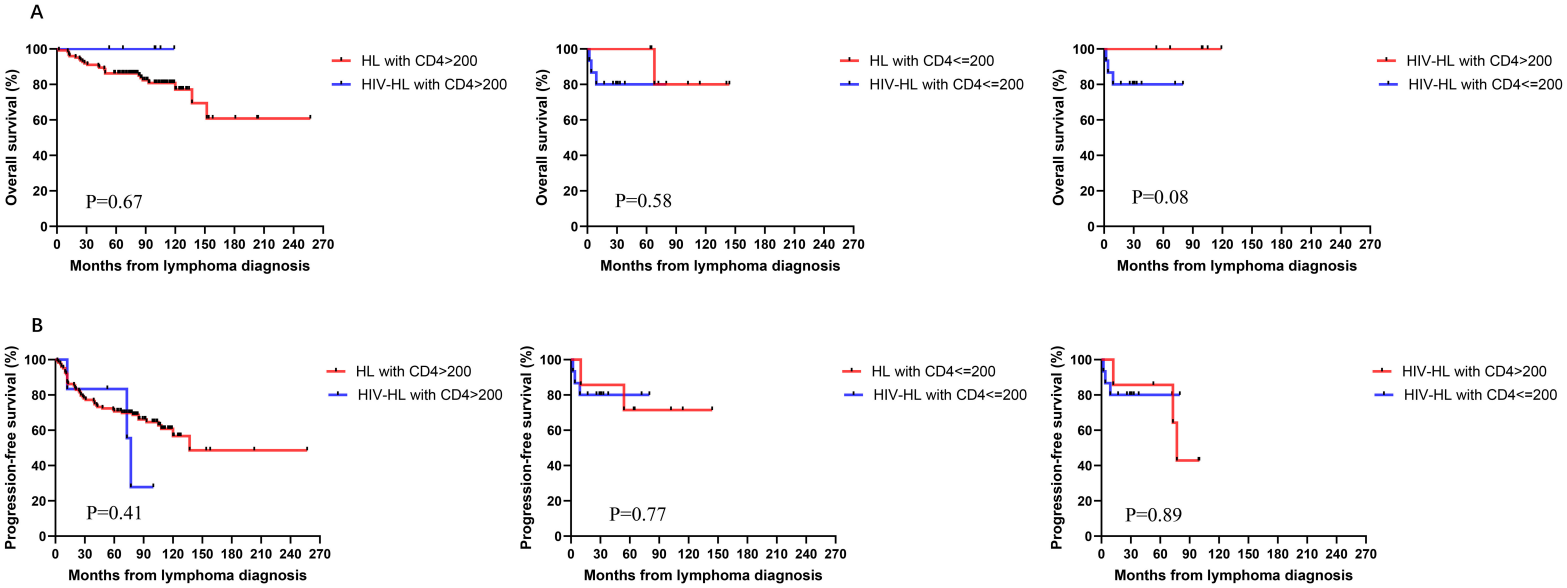
Similar OS **(A)** and PFS **(B)** between HIV-HL and HL patients, regardless of whether CD4>200 cells/ul, CD4≤200 cells/ul. HIV-HL had similar OS **(A)** and PFS **(B)** regardless of whether CD4 T cells were recovered.

**Figure 6:**
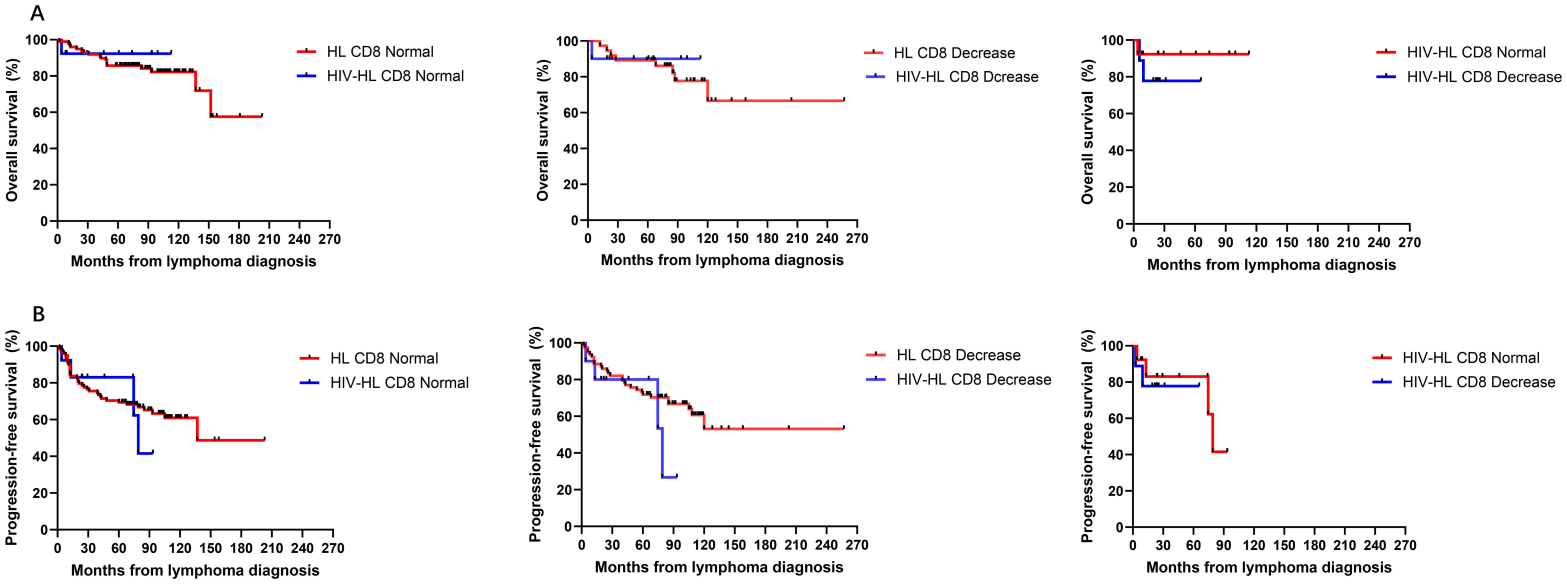
Similar OS **(A)** and PFS **(B)** between HIV-HL and HL patients, regardless of whether CD8 normal or decrease. HIV-HL had similar OS **(A)** and PFS **(B)** regardless of whether CD8 normal or decrease.

**Figure 7:**
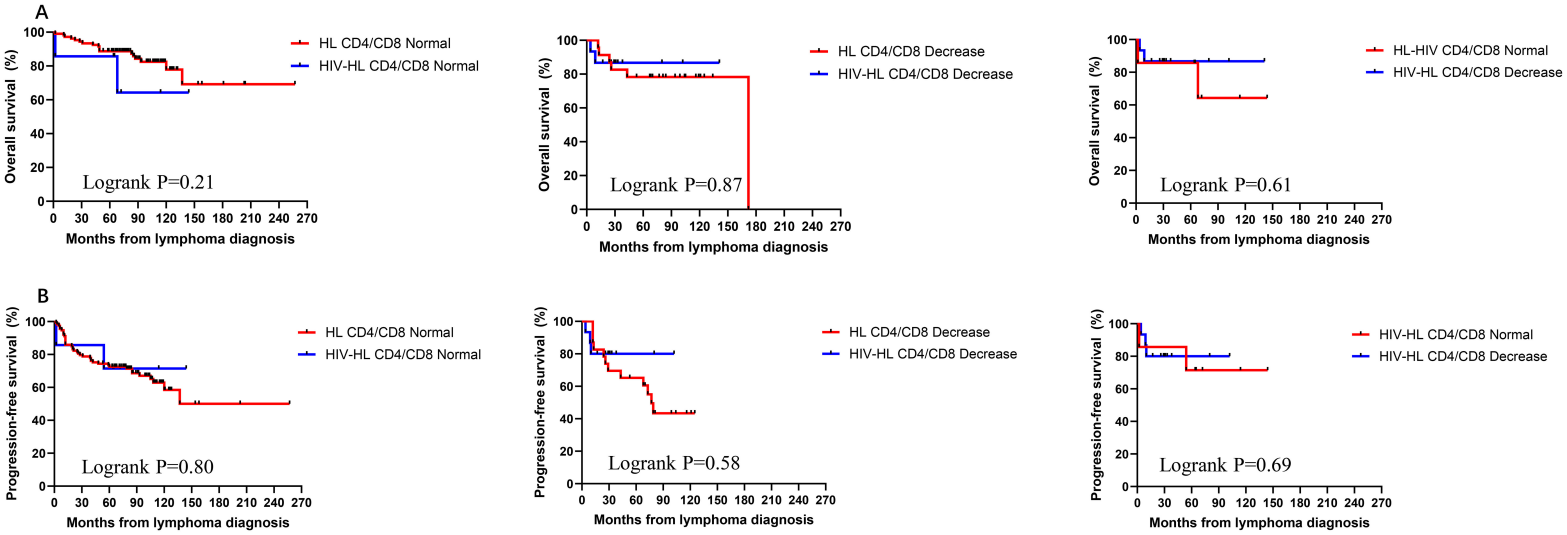
Similar OS **(A)** and PFS **(B)** between HIV-HL and HL patients, regardless of whether CD4/CD8 nomal or decrease. HIV-HL had similar OS **(A)** and PFS **(B)** regardless of whether CD4/CD8 nomal or decrease.

To investigate the impact of immune function on prognosis, we stratified all HL patients into two groups using a CD4 count threshold of 200 cells/μL (CD4 ≤ 200 vs CD4>200). Our results demonstrated no statistically significant differences in OS and PFS between CD4 ≤200 and >200 groups for both HIV-affected and non-HIV-affected HL patients (all P>0.05) (**Figure 5**). Subsequently, we examined the prognostic effect of CD8 counts (normal level: 360–1250 cells/μL) in HL patients. The analysis revealed that reduced CD8 levels did not significantly affect PFS or OS in either HIV-HL or conventional HL cases (shown in **Figure 6**) (all the P>0.05). Finally, we compared prognostic outcomes between patients with low versus normal CD4/CD8 ratios (0.71-2.87). The data indicated that CD4/CD8 ratio status had no significant influence on OS or PFS in either HIV-positive or HIV-negative HL cohorts (all P>0.05). (**Figure 7**).

### Prognostic factors

Our findings indicate that immune function status does not significantly affect HL prognosis. To further identify prognostic factors in HL, we performed both univariate and multivariate regression analyses. Univariate analysis showed that age ≥60 (p=0.028), advanced stage (p=0.012), B symptoms (p=0.001), poor ECOG-PS (p=0.013), elevated β2-MG (p=0.005), decreased WBC (p=0.006), and decreased neutrophils (p=0.024) were predictive of worse PFS. Age ≥60 (p=0.006), advanced stage (p=0.005), B symptoms (p=0.006), poor ECOG-PS (p<0.001), elevated β2-MG (p=0.001), decreased WBC (p=0.001), decreased lymphocytes (p=0.02), and decreased neutrophils (p=0.008) were predictive of worse OS. Cox multivariate analysis showed that decreased WBC were independent risk factors for adverse prognosis based on PFS (p=0.041). Poor ECOG-PS, elevated β2-MG, and decreased WBC were independent risk factors for adverse prognosis based on OS (p=0.009, p=0.035, p=0.045, respectively) (**Table 4**).

**Table 4:** Prognostic factor analysis for progression free survival and overall survival.

Variables	Progression-free survival				Overall survival			
	Univariate		Multivariate		Univariate		Multivariate	
	HR (95% CI)	p	HR (95% CI)	p	HR (95% CI)	p	HR (95% CI)	p
Gender (F/M)	1.052 (0.585-1.893)	0.865			0.612 (0.289-1.295)	0.199		
Age (<60/≥60)	1.891 (1.073-3.333)	0.028	1.534 (0.852-2.761)	0.153	2.822 (1.352-5.891)	0.006	2.102 (0.985-4.489)	0.055
Stage (I-II/III-IV)	2.120 (1.180-3.810)	0.012	1.330 (0.666-2.655)	0.418	3.949 (1.504-10.370)	0.005	2.509 (0.827-7.615)	0.104
B symptoms	2.627 (1.523-4.533)	0.001	1.823 (0.943-3.522)	0.074	2.856 (1.354-6.022)	0.006	1.079 (0.429-2.711)	0.872
ECOG (0-1/2-4)	3.254 (1.278-8.282)	0.013	1.695 (0.636-4.516)	0.291	8.772 (3.201-24.040)	<0.001	4.289 (1.446-12.723)	0.009
HIV positive	1.072 (0.454-2.532)	0.873			1.130 (0.336-3.795)	0.843		
EBV positive	1.141 (0.658-1.978)	0.638			0.792 (0.365-1.717)	0.555		
CD4 (<200/≥200)	0.997 (0.424-2.340)	0.994			0.538 (0.203-1.430)	0.214		
CD4/CD8	1.629 (0.900-2.948)	0.107			1.732 (0.785-3.820)	0.174		
β2-MG	2.293 (1.281-4.104)	0.005	1.487 (0.769-2.876)	0.239	5.534 (2.094-14.626)	0.001	3.122 (1.082-9.007)	0.035
LDH	1.322 (0.744-2.348)	0.342			1.335 (0.604-2.952)	0.475		
BM involvement	2.185 (0.922-5.180)	0.076			2.689 (0.921-7.848)	0.070		
Bulky tumor	0.230 (0.032-1.664)	0.145			0.502 (0.068-3.705)	0.499		
WBC	2.277 (1.265-4.100)	0.006	1.982 (0.946-4.152)	0.041	3.630 (1.727-7.632)	0.001	2.612 (1.021-6.678)	0.045
Lymphocyte	1.075 (0.624-1.852)	0.794			2.418 (1.153-5.074)	0.020	1.920 (0.889-4.416)	0.097
Neutrophils	2.394 (1.122-5.108)	0.024	1.377 (0.531-3.569)	0.510	3.376 (1.366-8.345)	0.008	1.442 (0.473-4.393)	0.520
PLT	1.439 (0.724-2.861)	0.299			1.828 (0.773-4.322)	1.170		

## Discussion

In this retrospective analysis of patients treated for HIV-HL in China, we found that most PLWH had more advanced stage, higher tumor burden, and a more decreased immune function. CD4 cell count was connected with decreased immune function and advanced tumor burden. HIV-positive HL patients had similar outcomes to those without HIV. However, decreased immune function was not related to poor outcomes.

Consistent with prior reports (12), HIV-HL patients in our cohort demonstrated a male predominance, mirroring the gender distribution observed in HIV-negative HL populations. While classical HL typically peaks in adolescence, HIV-HL predominantly affects older adults (13–15). Still, in China, no significant difference was observed in the median age of HIV-HL and HIV-negative HL patients. Mixed cellularity subtype (MC-HL) emerged as the most frequent histology in HIV-HL cases, accounting for 17/19 (89%) of Chinese cases in our series—a finding consistent with global patterns (16).

Most of the HIV-HL patients presented immunocompromised and more aggressive characteristics, higher tumor burden at diagnosis, including advanced-stage presentation (Ann Arbor stage III/IV), extranidal involvement, and B symptoms. These findings are consistent with previous reports (17). The exact mechanism underlying the more aggressive characteristics of HIV-HL compared to HIV-negative cases is not fully understood. It may be principally attributed to later diagnosis and incomplete immune reconstitution (median CD4+ count: 110 cells/μL despite cART). 63.2% of patients were diagnosed with HIV previous to their HL diagnosis in our study. The immune microenvironment in HIV-positive HL patients showed less CD4 and CD8 and lower GZMB secretion by T cells than HIV-negative patients. Notably, patients with suboptimal CD4+ T-cell recovery (persistent counts <200 cells/μL) demonstrated greater susceptibility to R-S cell development even during antiretroviral therapy (18), suggesting the critical role of sustained immunodeficiency in lymphomagenesis.

To evaluate the impact of low CD4^+^ counts in HIV-HL, we compared patients using a CD4^+^ threshold of 200 cells/μL. Consistent with prior studies, HIV-positive patients with lower CD4^+^ counts exhibited higher rates of advanced tumor burden (19). Our analysis also revealed an interesting finding that lower CD4 count was correlated with bulky disease and B symptoms. Supporting this, we observed diminished GZMB expression in bulky HIV-HL tumors. This correlation is likely attributable to impaired immune surveillance facilitating tumor proliferation and dissemination (20). Decreased CD4 level may contribute to suppression of effector T cell and attenuate elimination of malignant cells (19). This would contribute to tumor proliferation. Concurrent cytokine imbalance—particularly STAT3-activating IL-6/IL-10 secreted by activated monocytes—represents an additional driver of bulky disease (21). Notably, elevated IL-2Rα and IL-6 levels were detected in our HL-HIV patients, with further increases in those with bulky disease. Sustained pro-inflammatory cytokine production (notably IL-6, TNF-α, and IL-2R) induces CD4^+^ T-cell exhaustion and accelerates tumor progression (22). The enrichment impact of gene alterations on bulky disease shows that the PI3K/AKT pathway and EBV infection were involved in lymphomagenesis. This might be explained by parts of immune cell function impairment contributing to lymphomagenesis through multiple mechanisms (7–9).

However, our analysis found no significant prognostic difference attributable to baseline immunodeficiency status. This observation aligns with contemporary studies demonstrating that, with the advent of cART, HIV-HL patients receiving standard HL treatment regimens achieve better or similar outcomes as HL in the general population when applying the same treatment (10, 11, 16). A retrospective study revealed the five-year OS was 81% and 88% for HIV-positive and HIV-negative patients treated with ABVD (16). Similarly, in our study, patients treated with ABVD had similar OS and PFS between HIV-positive and HIV-negative instances, with five-year OS of 86.4% and 86.8%, respectively.

To better understand the impact of immune dysfunction on survival outcomes of HL and HIV-HL patients, we investigated the prognostic value of CD4+ T-cell counts, CD8+ T-cell counts, and the CD4/CD8 ratio. As demonstrated by Sparano JA et al., CD4 counts showed no significant association with CR rates, PFS, or OS in HIV-associated diffuse large B-cell lymphoma (DLBCL) patients (23). Our data revealed that CD4, CD8, and CD4/CD8 ratio status had no significant influence on OS or PFS in either HIV-positive or HIV-negative HL cohorts. Immunological dysfunction does not independently impact survival outcomes in the contemporary treatment era. Survival improvements in immunosuppressed patients are contingent upon the delivery of full-dose chemotherapy intensity and cART therapy (20, 23). This supports the use of identical treatment protocols for HIV-positive and HIV-negative patients.

This phenomenon is partially attributable to the efficacy of cART. Studies demonstrate that, in the cART era, cART treatment partly restores immune function to enable effective delivery of lymphoma-directed therapies (18). The survival of RS cells within the tumor microenvironment exhibits greater dependence on the PD-1/PD-L1 axis or EBV infection signaling than on CD4^+^ T-cell abundance (24). Furthermore, the immune cells, beyond just CD4 T cells, lead to a markedly altered tumor microenvironment of HIV-HL contrasted to HL without HIV infection (14, 25). Compensatory immune pathways, particularly CD8^+^ T-cell cytotoxicity and NK cell-mediated antibody-dependent cellular cytotoxicity (ADCC), may overcome CD4^+^ deficiency to contribute to tumor regression (26).

However, lower CD4 T cell counts were associated with increased HL risks, even in patients receiving effective cART (27). In the cART era, HL patients usually experience a moderate decrease in median CD4 count (28, 29). It has been speculated that this fact could be because a certain number of CD4 cells are needed to facilitate the micro-environment development and proliferation of HRS cells. In turn, HRS cells produce many cytokines and chemokines, resulting in an infux of activated CD4 cells (1, 28). However, very low CD4 counts would lead to an impairment of these mechanisms and, hence, to a worse condition for the development of HL in severely immunosuppressed PLWH (28).

There are also some limitations. Firstly, this study did not include many HIV-positive HL patients, which may increase the difficulty of making meaningful statistical results and result in potential limitations in statistical power. The risk of Type II errors should also be considered. Secondly, it was a retrospective study and therefore potential for selection bias should be considered. We were not able to obtain all immune cell information on tumors due to a lack of data. Therefore, the immune function analysis was not comprehensive enough. Furthermore, we did not address the HIV-RNA level or EBV coinfection in our analysis, which could further illuminate the effect of HIV on presentation and survival of HL.

## Conclusions

The characteristics of this lymphoma in the HIV setting often present with more aggressive clinical features in HIV-positive HL, while outcomes are similar to those seen in HIV-negative HL patients. Impaired immune function may contribute to an increased tumor burden through multiple mechanisms. However, immune dysfunction was not associated with outcomes in both HIV-positive and negative HL patients. HL treatment strategies need not be modified solely based on HIV status.

## Data Availability

The raw data supporting the conclusions of this article will be made available by the authors, without undue reservation.
